# Visual Semantic Landmark-Based Robust Mapping and Localization for Autonomous Indoor Parking

**DOI:** 10.3390/s19010161

**Published:** 2019-01-04

**Authors:** Junqiao Zhao, Yewei Huang, Xudong He, Shaoming Zhang, Chen Ye, Tiantian Feng, Lu Xiong

**Affiliations:** 1 MOE Key Laboratory of Embedded System and Service Computing, and the Department of Computer Science and Technology, School of Electronics and Information Engineering, Tongji University, 4800 Caoan Road, Shanghai 201804, China; zhaojunqiao@tongji.edu.cn (J.Z.); hexudong@tongji.edu.cn (X.H.); yechen@tongji.edu.cn (C.Y.); 2 School of Surveying and Geo-Informatics, Tongji University, 1239 Siping Road, Shanghai 200092, China; huangyewei@tongji.edu.cn (Y.H.); zhangshaoming@tongji.edu.cn (S.Z.); 3 School of Automotive Studies, Tongji University, 4800 Caoan Road, Shanghai 201804, China; xiong_lu@tongji.edu.cn

**Keywords:** autonomous driving, semantic landmark, parking lot, robust SLAM

## Abstract

Autonomous parking in an indoor parking lot without human intervention is one of the most demanded and challenging tasks of autonomous driving systems. The key to this task is precise real-time indoor localization. However, state-of-the-art low-level visual feature-based simultaneous localization and mapping systems (VSLAM) suffer in monotonous or texture-less scenes and under poor illumination or dynamic conditions. Additionally, low-level feature-based mapping results are hard for human beings to use directly. In this paper, we propose a semantic landmark-based robust VSLAM for real-time localization of autonomous vehicles in indoor parking lots. The parking slots are extracted as meaningful landmarks and enriched with confidence levels. We then propose a robust optimization framework to solve the aliasing problem of semantic landmarks by dynamically eliminating suboptimal constraints in the pose graph and correcting erroneous parking slots associations. As a result, a semantic map of the parking lot, which can be used by both autonomous driving systems and human beings, is established automatically and robustly. We evaluated the real-time localization performance using multiple autonomous vehicles, and an repeatability of 0.3 m track tracing was achieved at a 10 kph of autonomous driving.

## 1. Introduction

Autonomous driving has seen considerable progress in recent years. Researchers have made breakthroughs in several challenging fields, including obstacle detection, real-time motion planning and high-precision localization (many based on differential global navigation satellite system (GNSS)). Self-driving cars have recently been shown to drive safely in urban and suburban areas (https://waymo.com). However, parking in a large indoor parking lot without human intervention remains an unsolved problem, especially with only budget visual sensors. One critical reason is the lack of robust high-precision localization in GNSS-denied areas. Traditional indoor localization methods include the inertial sensor-based dead-reckoning systems [1] and the wireless-based localization systems, such as WiFi, Bluetooth, RFID or Ultra-Wide Band (UWB) [2]. However, dead-reckoning systems could not relocalize a vehicle and inertial sensors tend to drift without the updating from the GNSS system. The wireless-based systems suffer from occlusion in complex indoor environments and decay as the user’s distance to the signal sources increases. Therefore, a considerable number of stations are required for stability and acceptable precision [3]. Laser-based simultaneous localization and mapping (SLAM) systems are eligible for the localization of unmanned vehicles in environments such as factories and warehouses [4]. However, this method requires expensive LiDAR sensors and is characterized by an enormous data volume and high computational cost. As a result, visual SLAM (VSLAM), which is based on low-cost cameras, has become one of the most demanded localization methods.

**Figure 1:**
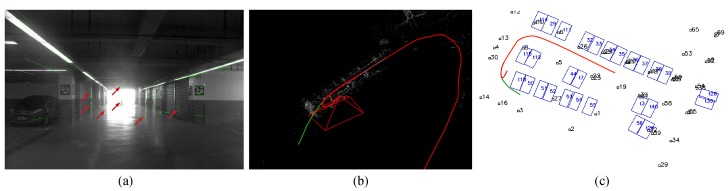
(**a**) Wrongly matched feature pairs (indicated by red arrows) interfere with the initialization of ORB-SLAM2. (**b**,**c**) HorizonAD stereo DSO result (red) compared with real vehicle trajectory (green) from both the perspective and top-down view.

Traditional visual feature-based VSLAM is effective in texture-rich environments [5]. Nevertheless, as shown in Figure 1a, VSLAM can easily fail in monotonously textured scenes, such as an indoor parking lot. Grimmett et al. [6] first adopted sparse feature-based SLAM with panoramic images to localize a car in a parking lot. However, the extracted BRISK features can be unstable when the ground floor is stained with tire markings or water spots. The distortion presented in the stitched images can also disturb the feature extraction.

Direct methods estimate camera poses based on the photometric error derived from the whole image and thus are more robust than sparse methods in textureless areas [7,8]. HorizonAD applied such a method for indoor parking (https://github.com/HorizonAD/stereo_dso). However, these methods often require high frame rates and are susceptible to global illumination changes, which restricts their usage in unevenly illuminated indoor parking lots [9]. Figure 1b shows the results of this method applied to our testing parking lot. Only a small part of the trajectory can be estimated before drastic drifting. More importantly, the re-localization based on a pre-built dense map is not trivial since illumination can vary when revisiting. Therefore, most direct VSLAM methods are rather visual odometries [7]. As a result, stable and legible visual landmarks that are immune to various illumination conditions are demanded.

**Figure 2:**
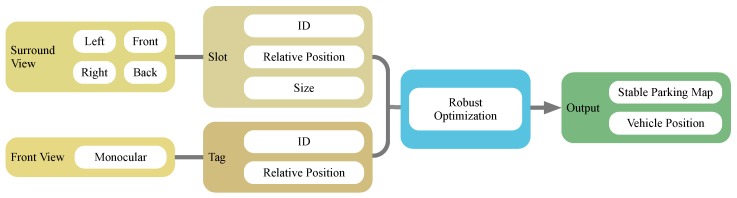
Pipeline of the method: Slots’ IDs, positions and sizes are detect from the surround view image fused by fisheye frames. Tags’ positions and IDs are detected from the front view image. Slots are then roughly matched according to their poses and ID and a robust back-end is also introduced to detect slot matching outliers. The final output is a stable parking map with the real-time vehicle position.

As a typical kind of semantic landmark in parking lots, the parking slot is one of the focuses for researchers [6,10,11]. Recently, deep learning-based methods have shown their capability for accurate and robust detection of such meaningful objects [12]. Inspired by these methods, we present a robust VSLAM system based on the recognition of high-level landmarks for parking, i.e., parking slots. The whole pipeline is shown in Figure 2. The parking slots are detected in the surround view composed of four fish-eye cameras. Both the geometry of the slots, i.e., the position and the size, and the IDs of the slots (which are optional) are extracted and treated as landmarks in the proposed SLAM system. To support localization in slot-lack area such as passways, we introduce visual fiducial tags detected from the front view camera for improving the overall accuracy and robustness. Their numbers and configurations are further analyzed. Faced with the visual aliasing problem of parking slot landmarks, we propose a robust strategy that incorporates slot confidence level to semantically detect and eliminate outliers in the optimization stage.

Finally, a two-dimensional map of parking slots, which is distinguished from the traditional visual feature-based maps in terms of its stability, re-usability, lightweight and human readability, can be robustly established. Our system is implemented on low-cost autonomous driving vehicles and tested in real parking lots.

This paper is a substantial extension of our previous paper [13]. The main contributions are the following:

- We design and implement a low-cost and robust visual-based SLAM system using a typical visual landmark of parking slots with aid from a limited number of visual fiducial tags, which is immune to monotonous texture, varying illumination and dynamic conditions;
- We propose a robust SLAM back-end approach to associate parking slots considering the confidence level of the landmarks;
- We analyse the effectiveness and arrangement strategy of visual fiducial tags in a typical indoor parking lot.

The rest of this paper is organized as follows. Section 2 summarizes previous research on VSLAM, semantic SLAM, and robust SLAM methods. Section 3 introduces our landmark-based indoor parking SLAM method. Meanwhile, our semantic landmark detection and robust outlier elimination strategy are detailed in Section 3.1 and Section 3.2. Section 4 presents the experiments and analysis, while the potential improvements of our methods are discussed in Section 5.

## 2. Related Work

The related works are separated into three parts: the VSLAM, the semantic landmark-based VSLAM, and the robust SLAM methods.

### 2.1. Visual SLAM

SLAM has long been a classic topic in the field of robotics [14] and has become a popular research direction in autonomous driving since many drivable areas are GNSS denied, such as indoor parking lots and urban roads covered by tree branches or beside skyscrapers [15]. VSLAM, which is based on budget vision sensors, is preferred by many car manufacturers who have already equipped multiple cameras on their production cars. Generally, traditional VSLAM methods fall into two groups, feature-based methods (indirect methods) and image-based methods (direct methods).

Feature-based methods rely on sparse visual features extracted from images, i.e., corners, blobs. Features are fast and accurate to extract, and invariant descriptors make the features robust against certain viewpoint and scale changes. As a result, in a VSLAM system, frames are matched based on these features for estimating local transformations of camera poses. These features are also packed to facilitate data associations for detecting loops [5].

The optimization back-end evolved from filter-based methods [16] to factor graph-based methods [17]. As one of example, ORB-SLAM offers a stable and efficient graph-based VSLAM system based on ORB features [5]. The system consists of several parallel operating tasks, such as keyframe detection and tracking based on feature matching, Bag-of-Words empowered fast loop closure detection, and local and global bundler adjustment (BA). This system performs well in texture-rich indoor and outdoor environments in real time.

Since feature-based methods are capable only of creating a sparse map, they cannot be directly used in applications where full reconstruction is demanded, e.g., Argument Reality (AR) or structure from motion (SfM). To satisfy those applications, direct methods based on the photometric error, which uses all image pixels, have been proposed [7]. Compared with feature-based methods, direct methods aim to estimate pixelwise coordinates and can output a dense or semi-dense point cloud in real time. However, in practice, direct methods require a high rate of overlap between sequential frames. Moreover, a high frame rate is also required since brightness consistency is crucial for estimating the depth accurately [18]. Semi-direct Visual Odometry (SVO) [8] and Direct Sparse Odometry (DSO) [19] combine the advantages of feature-based methods and direct methods. However, these odometric methods, which lack loop closure detection, drift as time increases and cannot easily re-localize a vehicle.

### 2.2. Semantic Landmark-Based SLAM

The low-level descriptions of images adopted by the above methods are vulnerable to textureless scenes, and such descriptions are also difficult to adapt to dynamic changes in scenes [14]. Moreover, feature-based methods do not incorporate humanly understandable meanings (semantics) associated with landmarks into the method, which now is recognized to be crucial for strengthening the descriptive power of landmarks and constructing a human-readable map [14]. On the contrary, the human cognitive system relies on abstractive object-based landmarks for localization and navigation. These landmarks are meaningful and robust against different backgrounds, shapes, perspectives, illumination and even occlusions. Object detection in images has obtained unprecedented precision empowered by the deep learning-based methods [20]. Therefore, object-based semantic SLAM (in short, semantic SLAM) has become one of the forefronts of SLAM.

SLAM++ [21] was the first object-based SLAM method to employ furniture as semantic landmarks. Objects were segmented from RGB-D observations and were matched using Iterative Closest Point fitting (ICP). The back-end was the popular graph-based optimization, but semantics were minimally helpful in the optimization stage.

In [22], shop names and facades were recognized and combined as landmarks for location inferencing based on the 3D floor plan of an indoor shopping space. Li and Belaroussi [23] added semantic labels to an LSD-SLAM framework to construct a dense map with classes attached to geometric entities. Gálvez-López et al. [24] first incorporated visually detected objects into the graph SLAM. The pre-built 3D object database and ORB-feature were employed for object detection. However, the feature-based object description cannot be used in textureless scenes. Mccormac et al. [25] employed semantic labels in the RGB-D SLAM framework to aid loop closure. Sünderhauf et al. [26] detected and segmented meaningful objects from RGB-D data and archived the semantic mapping in office scenarios. However, this method relied heavily on ORB-SLAM.

Grimmett et al. [6] and Himstedt and Maehle [11] reconstructed the metric map and the semantic map of an indoor driving scene, i.e., a parking lot and a warehouse, but they adopted traditional visual feature-based or grid-based SLAM methods. Object-based landmarks were not employed. The recently proposed CubeSLAM [27] adopted both traditional feature points and 3D cuboid to represent objects as joint landmarks, but the data association still relied on feature point matching.

To the best of our knowledge, there has been little application of object-based SLAM for practical usage, especially for autonomous vehicles.

### 2.3. Robust SLAM

Although semantic landmarks are robust and consistent for observation, the association between them is more difficult than the association between visual features because of their equal representations as objects.

To reduce the probable erroneous associations, the robust SLAM methods were proposed [28,29,30]. These methods can be performed either in the optimization stage, i.e., switchable constrains (SC) [28], max-mixture (MM) [29] or as a middle layer before optimization i.e., Realizing, Reversing, Recovering (RRR) [30], Graphtinker (GTK) [31].

SC and MM adopted multi-model constraints in the graph and attempted to find the best constraint that conforms to the global optimal. The topologies of the graphs in both methods are modifiable instead of fixed. Although the inferior correct constraints were eliminated, which may result in an undesirable sparse graph, these methods are flexible and are convenient to integrate into optimization. RRR and GTK detected outliers by maintaining only consistent loop closures before optimization and can operate collaboratively with previous methods. GTK can even introduce artificial constraints to robustify the final graph and improve the results when detained with a high portion of outliers. However, both methods have slow computation speed compared to SC and MM [31,32].

Methods that optimize the distribution of data associations together with the graph were recently proposed, mainly for object-based SLAM [33,34]. However, these methods were complicated to be integrated into existing optimization frameworks and the performance is still inadequate for real-time usage.

In summary, existing VSLAM methods generally do not perform robustly in textureless areas such as an indoor parking lot. Therefore, more descriptive landmarks, especially landmarks attached to semantics, should be used. However, robust data association for these semantic landmarks should be proposed.

## 3. Approach

**Figure 3:**
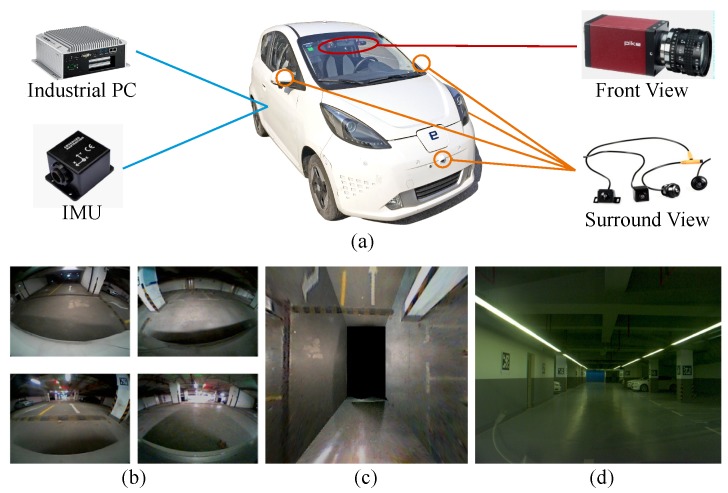
The low-cost sensors configuration used in our proposed system (**a**) Four fisheye cameras, two monocular cameras, a low-cost inertial navigator and a computer are equipped. (**b**) The captured images from four fisheye cameras. (**c**) Fused bird’s-eye view image. (**d**) The image from one of the monocular camera.

As shown in Figure 3a, our semantic VSLAM system is based on images captured by four fisheye cameras and one monocular front view camera, which have now become a standard configuration in many production vehicles. The four fisheye cameras are fixed at two reflectors and at the front and rear bumpers to create a surround-view system. A bird’s-eye view image is then fused from the surround-view inputs after intrinsic and extrinsic calibration, as shown by Figure 3c) Parking slots can be detected in the bird’s-eye view image, which indicates ground textures. We use a monocular camera installed to the left of the rear-view mirror to capture front-view scenes (Figure 3d). The steering wheel angle, as well as the vehicle speed and heading direction collected by a budget inertial measurement unit (IMU), Xsens MTi-3 AHRS, compose a dead-reckoning subsystem. The steering wheel angle and the heading direction are first fused by a Kalman filter, and the fused heading is integrated with the vehicle speed to estimate the 3-DoF translation and rotation.

**Figure 4:**
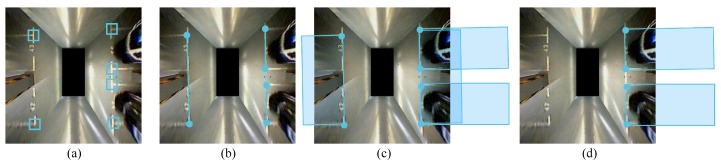
This figure illustrates how parking slots are detected. Corner patterns are detected (**a**) and assembled (**b**), which enables the initial parking slot hypothesis (**c**). (**d**) false positives are discarded by constraints based on prior knowledge of slot shapes.

Our parking slot detector is based on [12], in which the corner points of parking slots are detected and assembled (Figure 4). Although the CNN-based method is capable of detecting most types of corner points in a rapid and robust manner, only part of the slot is visible due to the limited visual field of the surround vision system, as Figure 4 shows. As a result, the shape of the parking slot can only be guessed initially. Furthermore, the ID of each parking slot is detected for facilitating data association between parking slots, which will be elaborated in Section 3.1.

Another type of landmark used in our system is the visual fiducial tag. Fiducial tags are introduced as an aid for the constancy of localization since few parking slots are detected near the entrances and exits. In our study, we found fiducial tags may not be entirely prohibited, but their number can be limited to a small amount. We select AprilTags as the fiducial tags for their robustness and high efficiency [35].

### 3.1. Semantic Landmark Recognition

The semantic parking slot recognition procedure is twofold: detecting the slot position and determining the slot ID, both of which are detailed in the following subsection. Moreover, the measurement of visual fiducial tags is also mentioned.

#### 3.1.1. Learning-Based Parking Slot Detection

We adopt the method proposed by Li et al. [12] to detect parking slots. The method is an AdaBoost-based slot detection method that detects parking slots from calibrated bird’s-eye view images. Slot detection is achieved by first recognizing the corner patterns from the image. Figure 4a shows examples of detected corner patterns. Since not all the corners of a parking slot may be fully observable, the parking slots are estimated according to their entrance lines (Figure 4b), which are determined by the configuration of patterns. Several constraints are applied to robustify the detection results: entrance-line candidates that contain more than two corner patterns are removed to avoid repeated detection. Extremely large or small candidates are also discarded because all slots are approximately the predefined size. Then, each slot is parametrized by a pose (direction and center position) and inserted into the global map as an entirety. Moreover, our framework can also be extended to optimize the shapes, as done by Yang and Scherer [27].

#### 3.1.2. CNN-Based Slot ID Recognition

**Figure 5:**
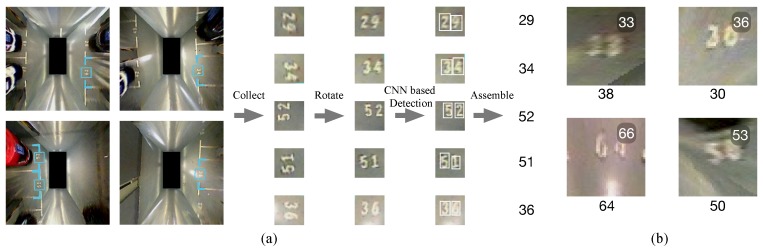
The slot ID detection method: (**a**) the slot ID detection pipeline, (**b**) examples of harsh image patches for ID detection. Digits under images are the true IDs, and the white digits shown at the top-right corner are the detection outputs.

ID of each parking slot can be used for the association of these semantic landmarks. We fine-tuned PVANet to detect each digit of the ID [36]. Figure 5a illustrates how the entrance line of a parking slot helps to roughly locate IDs and how slot IDs are detected. Image patches containing slot IDs are extracted and aligned according to the entrance line. Each digit is first detected as an individual and then assembled. During the training phase, we augmented the training sets by rotating the samples by random small angles (0°–5°) and created blurred and shaded samples. Unfortunately, due to the heavily distorted and blurred texture, especially in the corner of the surround view image (Figure 5b), even the sophisticated detection network could not achieve satisfactory performance. Therefore, our proposed robust back-end should be able to cope with the uncertainty, which will be detailed in Section 3.2.

#### 3.1.3. Visual Fiducial Tags

Recalling the goal of developing a robust localization system for practical autonomous driving and indoor parking, we introduced certain numbers of artificial landmarks, i.e., visual fiducial tags. One reason for the tags is to help the vehicle localize in entrances and corridors where few parking slots exist. The other is to introduce “faithful” constraints as anchors to the graph for robust optimization.

We adopted AprilTag [35] and its detection library (https://april.eecs.umich.edu/wiki/AprilTags). Moreover, the relative position between fiducial tags and the vehicle are solved by the Perspective-n-Point (PnP) model [37] in a fast and accurate way [13].

Other types of objects, such as signs or edges of pillars, could potentially be used as landmarks too. However, fiducial tags are easy and generic to implement and provide high accuracy and robustness to various illumination conditions. We will show later in this paper that only a limited number of fiducial tags, e.g., 3 to 4, are needed for highly precise localization inside a parking lot.

### 3.2. Semantic-Based Robust SLAM

**Figure 6:**
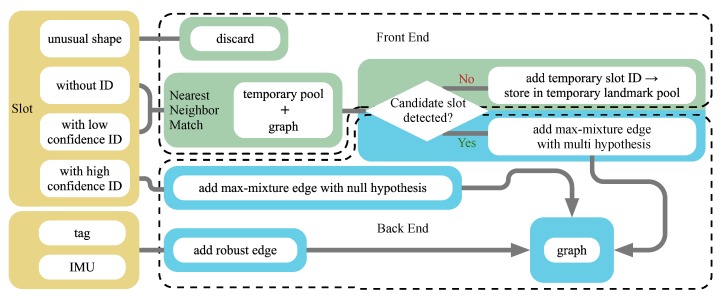
The overall pipeline of robust semantic SLAM. Green boxes are the front-end procedures and the back-end procedures are coloured in blue.

The fallible detection of parking slots and their IDs from low-quality bird’s-eye view images introduces erroneous data association, which is known to be hazardous to optimization in SLAM algorithms. Thus, it is crucial to employ a robust optimization strategy, where correct associations are ensured and incorrect associations are detected, discarded or even fixed. Our robust optimization pipeline includes a series of processes in both the front-end and the back-end (Figure 6).

#### 3.2.1. Front-End

In the front-end, the detected slots with abnormal size that are either too large or too small according to the standards are the first to be discarded. Then, the remaining slots are processed according to their slot IDs. Since the uncertainty present in the slot ID detection leads to uncertainty in slot observation associations, cases of multiple associations should be considered.

On one hand, detected slots with high confidence level are inserted into the graph or associated with previous observations directly. On the other hand, detected slots with low confidence level or undetectable IDs are associated with candidate slots by nearest-neighbour matching considering slot pose and shape.

The confidence level, $p_{ID}$, is given by the slot ID detector as the smallest probability of the digits of the detected ID. If the ID is not detected, $p_{ID}$ is decided by the slot detection accuracy.

(1) $$p_{ID}=\min\left\{p_{digit_{1}},p_{digit_{2}},p_{digit_{3}},\ldots \right\}$$

If no candidate slot is matched, the slot is regarded as a potential new slot and is stored in the temporary landmark pool and “lazily” (after being observed two or more times) inserted into the map. This “lazy” strategy efficiently avoids the impact of false positive slot detection on the map.

If one or more candidates are matched, the original slot observation and its candidate slot associations are initial multi-hypotheses. In other cases, slots with similar IDs generated by confusing digits, e.g., 1 and 7, and slots with partially detected IDs, e.g., only one of two digits is detected, offer associating alternatives for multi-hypothesis optimization. Moreover, IDs also act as a reference for correcting erroneous slot observations. When associating a slot observation to a candidate slot who has already been observed several times, their IDs are also compared. The ID of the former slot will be discarded if it is inconsistent with the latter one.

#### 3.2.2. Back-End

In the back-end, we adopt the popular g2o framework [38]. The classic graph optimization aims to solve a least-squares problem:

(2) $$\begin{array}{c} F\left(x\right)=\sum_{k\in C}e_{k}{\left(x_{k},z_{k}\right)}^{T}\Omega_{k}e_{k}\left(x_{k},z_{k}\right) \end{array}$$

(3) $$\begin{array}{c} x^{*}=\underset{x}{\arg\min}F\left(x\right) \end{array}$$

where uncertainties, denoted by error function $e_{k}$, are distributed according to each observation’s pre-designed and immutable information matrix $\Omega_{k}$. C denotes the set containing all edges, $x_{k}$ and $z_{k}$ are the state and observation vector of $k_{th}$ edge respectively.

However, the unimodal Gaussian adopted by classic graph-optimization cannot describe multiple hypotheses. Therefore, slot association, where ambiguity generally occurs, is added to the graph as multiple variable edges, expressed by the max-mixture model [32], with information matrices that are set switchable.

In the standard max-mixture method, the likelihood function of $x_{i}$ is expressed by a max-mixture of Gaussian distributions [29]:

(4) $$p\left(z_{i}^{j}|x_{i}\right)=\max_{j}w_{i}^{j}N\left(\mu_{i}^{j},{\Omega_{i}^{j}}^{-1}\right)$$

where $N(\mu_{i}^{j},{\Omega_{i}^{j}}^{-1})$ and $w_{i}^{j}$ denote the Gaussian distribution and weight for the $j^{\mathrm{th}}$ observation hypothesis $z_{i}^{j}$ generated in Section 3.2.1.

In this paper, $w_{i}^{j}$ and $\Omega_{i}^{j}$ are dynamically set according to the landmark’s confidence level $p_{ID}^{j}$:

(5) $$\begin{array}{c} w_{i}^{j}=b_{0}+b_{1}\cdot p_{ID}^{j}\;,\sum_{j=0\ldots N}w_{i}^{j}=1 \end{array}$$

(6) $$\begin{array}{c} \Omega_{i}^{j}=A_{0}+A_{1}\cdot p_{ID}^{j} \end{array}$$

where *N* is the total number of observation hypothesis and there is always a j=0 case denoting a false positive. The diagonal matrix $A_{0},\;A_{1}$ and parameter $b_{0},\;b_{1}$ are normalized coefficients and should be set considering both the slot detection and the tag detection accuracy.

The best observation hypothesis $z_{i}^{*}$ and its corresponding information matrix $\Omega_{i}^{*}$ are selected by the max operator. This offers flexibility to the graph, which helps to suppress the observation uncertainty. The max operator is indeed achieved by iterating through all hypotheses and selecting the best component that minimize the negative log of Equation (4):

(7) $$\begin{array}{cc} \left(z_{i}^{*},\Omega_{i}^{*}\right) & =\underset{j}{\arg\min}\left[-\log p\left(z_{i}^{j}|x_{i}\right)\right]\\ & =\underset{j}{\arg\min}\left[-\log w_{i}+\frac{\log|\Omega_{i}^{j}|}{2}+\frac{e_{i}{\left(x_{i},z_{i}^{j}\right)}^{T}\Omega_{i}^{j}e_{i}\left(x_{i},z_{i}^{j}\right)}{2}\right] \end{array}$$

Then, the original least-squares problem is transformed into a new mixed form containing both unimodal and max-mixture Gaussian distributions:

(8) $$\begin{array}{c} F\left(x\right)=\sum_{i\in C_{slot}}e_{i}{\left(x_{i},z_{i}^{*}\right)}^{T}\Omega_{i}^{*}e_{i}\left(x_{i},z_{i}^{*}\right)+\sum_{j\in C_{IMU,tag}}e_{j}{\left(x_{j},z_{j}\right)}^{T}\Omega_{j}e_{j}\left(x_{j},z_{j}\right) \end{array}$$

(9) $$\begin{array}{c} x^{*}=\underset{x}{\arg\min}F\left(x\right) \end{array}$$

Since $z_{i}^{*}$ and $\Omega_{i}^{*}$ have already been selected by Equation (7), the remaining steps are identical to those of classic graph optimization. Moreover, $z_{i}^{*}$ and $\Omega_{i}^{*}$ are re-evaluated dynamically in each optimization [29].

## 4. Experimental Analysis

**Figure 7:**
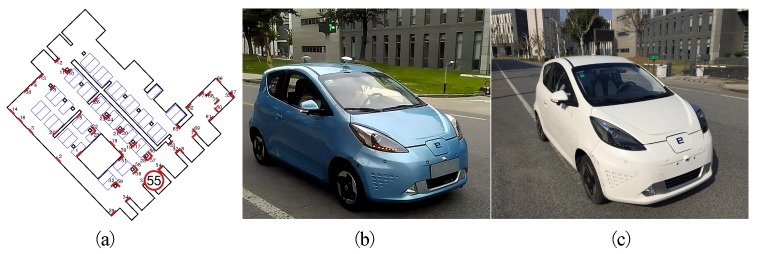
(**a**) A rough building plan of the parking lot with tags marked by red lines. (**b**,**c**) our test platforms.

In this section, we evaluate the results of the proposed method in the parking lot of the Telecoms building on Jiading Campus, Tongji University. The rough building plan of the parking lot, which covers an area of approximately 3000 square meters, is shown in Figure 7a. This parking lot is illuminated by dim lights, as shown by Figure 3. When facing the entrance, sunlight overexposes the front view cameras (Figure 1a). Other intruders include pedestrians and passing vehicles. The test platforms in Figure 7b are the two low-cost autonomous vehicles aiming for production prototype, which are equipped with previously mentioned sensors. The resolutions of the fisheye camera and the front-view camera are 640 × 480 and 1000 × 1000 respectively. The industrial computer is equipped with an i7 2.3 Ghz quad-core CPU and 8 GB memory and a Nvidia 1050Ti GPU with 4 GB memory. Both vehicles are able to drive autonomously.

We first tested and compared mapping results with different input measurements, i.e., Dead reckoning only, Parking slot-only and Parking slot aided by tags. Then the online localization performance is assessed. We further quantitatively analyzed the crucial influential factors of the configuration of the visual fiducial tags. The results show that only a few tags with high visibility and even spatial converge are required for robust mapping and localization.

### 4.1. Mapping with Semantic Landmarks

**Figure 8:**
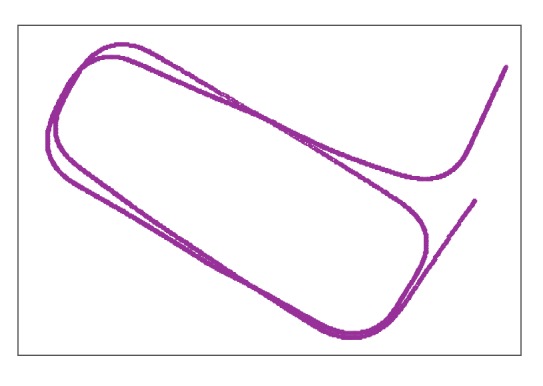
The drifting trajectory collected by the dead reckoning system at 5 kph.

The mapping process can be conducted either online or offline. During mapping, the transition between frames is estimated by the dead-reckoning system, as described in [13]. This dead-reckoning system drifts when the vehicle moves at a relatively low speed e.g., below 5 kph (shown by the trajectory in Figure 8).

#### 4.1.1. Parking Slot-Only Mapping

**Figure 9:**
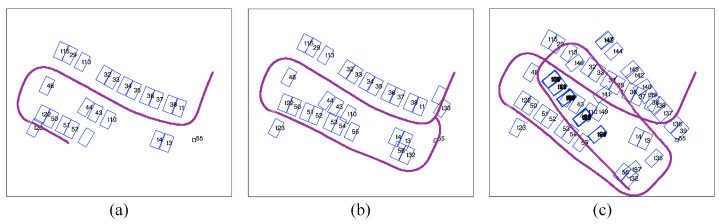
(**a**–**c**) are temporal results of mapping using observations of parking slots only at time stamp No. 1000, 2000 and 4000 respectively.

First, we test the performance of the parking slot-only mapping using the proposed method. The datasets are collected while the vehicle maintaining a speed of 3–5 kph by a human driver. Figure 9 shows the results of mapping without tag assistance at three time stamps. As time passes and the map expands, the error accumulates and distorts the map (shown from Figure 9a,b). However, the detected parking slots corrected the odometric error as compared to the trajectory in Figure 8. When the vehicle revisits a parking slot, large drifts prevent the new slot observation from been associated with the former (Figure 9b). Finally, the optimization falls into a local optimum, and both the vehicle trajectory and slot locations diverge considerably (Figure 9c). With purely the parking slot information, the mapping is difficult to converge even with the robust optimization method.

#### 4.1.2. Tag-Aided Parking Slot Mapping

**Figure 10:**
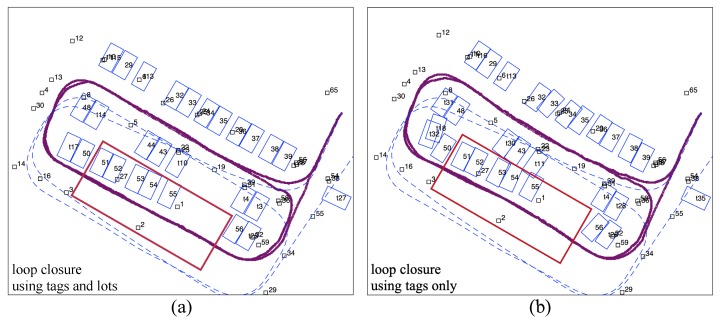
(**a**) Mapping result with loop closure using tags and slots, (**b**) Mapping result with loop closed using only tags. The dead-reckoning trajectory is shown by dashed lines in both figures.

To assist the parking slot-based SLAM, 60 A2-sized fiducial tags are placed in the parking lot to cover the slot-free area near the entrance and to guarantee a stable and credible observation for SLAM (as shown in Figure 10). During observations, tags that are 20 m or farther from the vehicle are discarded since the accuracy decreases as the tags become smaller, and the tags may even become unreadable in the image.

The mapping results of using combined tags and slots are compared with the results of using tags only. Figure 10a shows the mapping results using all tag and slot observations during optimization. Figure 10b shows the mapping results with tag observations only, and parking slots are added to the map only at their first sight. The comparison shows that with the assistance of tags, slot observations are correctly associated, and the drift caused by dead-reckoning is effectively abated. Nevertheless, in the area where tags are sparse (marked by the red square in Figure 10), observations of parking slots lead to crucial constraints for the optimization.

**Figure 11:**
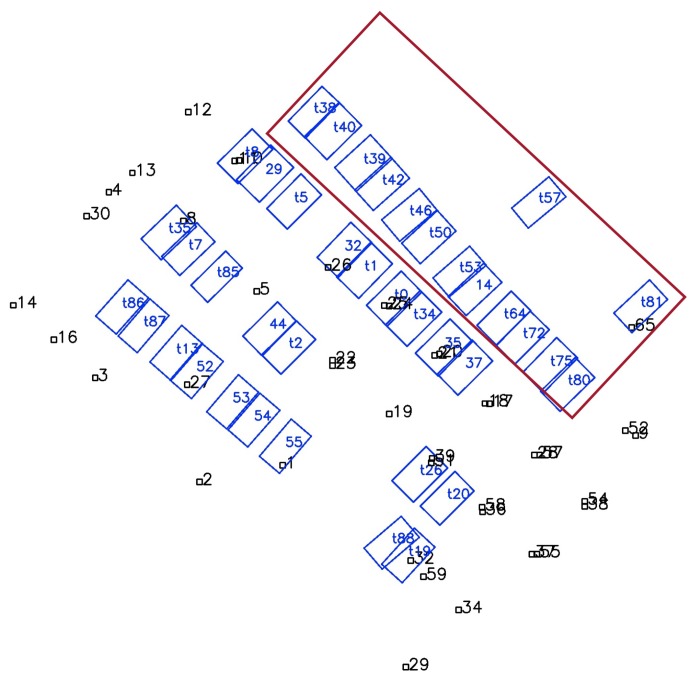
The mapping result of the whole parking lot, slot IDs starting with “t” are temporary slot IDs for slots where ID detection fails.

**Figure 12:**
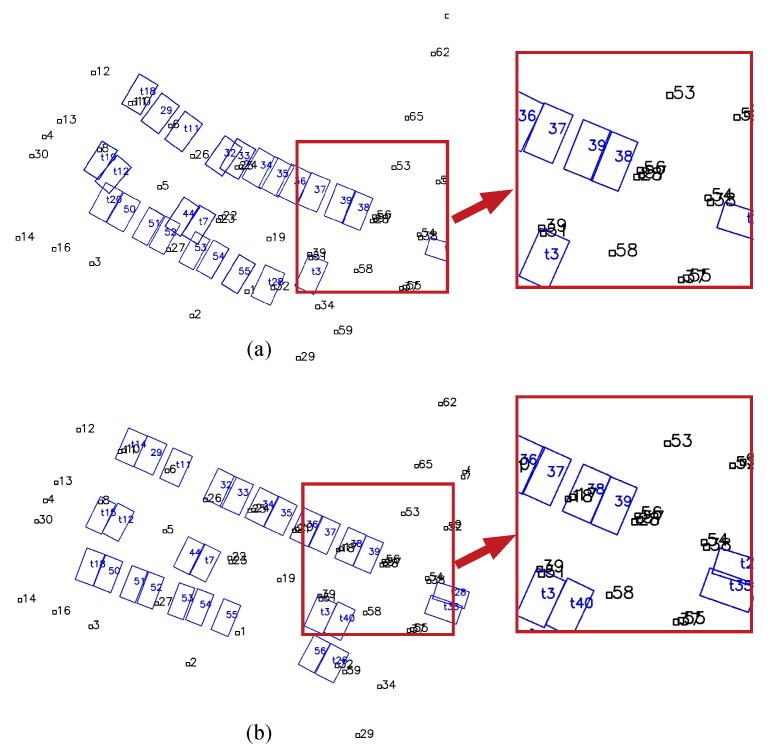
(**a**) Map optimized by the traditional graph-based method without a back-end error detection strategy, (**b**) map result optimized by the proposed robust SLAM method.

Figure 11 shows the map results of the whole experimental area using combined tags and slots. All the parking slots are successfully mapped. The undetected parking IDs minimally affect the mapping result, as shown by the slot with IDs starting with “t” in Figure 11. We compared the mapping results with and without the proposed robust method, and the results are shown in Figure 12. In Figure 12a, some slots are wrongly associated with others due to the incorrectly detected slot IDs, causing global ambiguity, e.g., slot No. 39 is wrongly recognized as No. 38 when firstly detected, and the IDs of the two slots reversed. In Figure 12b, the proposed robust method avoids this issue by choosing a more reasonable hypothesis.

### 4.2. Online Localization Performance

Since the ground truth of localization in an indoor space is difficult to obtain, we first evaluated the localization performance by measuring the “revisiting error” (repeatability) according to the revisiting of pre-recorded trajectory and poses. In addition, we then evaluated the mapping accuracy according to the measured distances between selected tag pairs.

**Figure 13:**
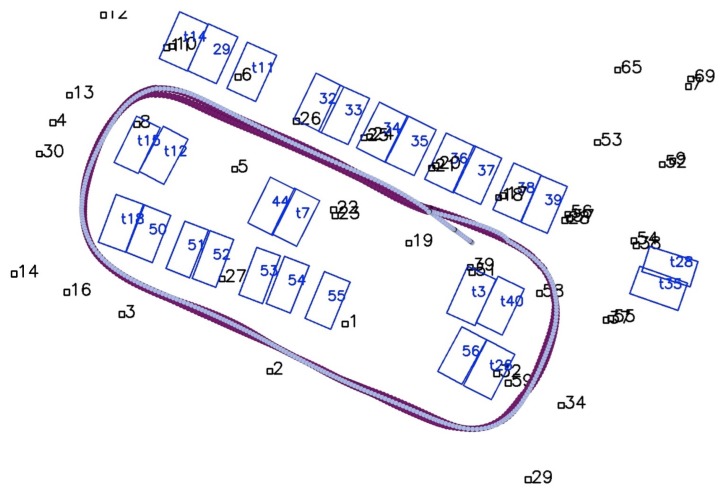
Comparison of the reference and automatic driving trajectories, where the blue trajectory is a human driving trajectory and the claret trajectory is the automatic driving trajectory.

The vehicle is first operated by a human driver to initialize the map. Once the map stabilizes, a reference trajectory is recorded. Then, the vehicle drives autonomously following this reference trajectory according to the real-time localization. The experiment is repeated for twelve times. The actual driving trajectory is recorded and compared with the reference trajectory. Figure 13 shows the trajectories of both manual (blue) and autonomous driving (claret). By measuring the deviation between actual driving trajectories and the reference trajectory (The deviation is calculated based on the distance between the closest points of two trajectories), the average revisiting error is 0.31 m, the minimum revisiting error is 0.125 m and the maximum revisiting error is 0.45 m. Both the two vehicles could autonomously follow the reference trajectory at the maximum speed of 10 kph without human intervening.

Because the previous dynamic revisiting error contains uncertainties from both the localization and the control system, thus, we further evaluated the localization precision by manually driving the vehicle repeatedly to revisit three pre-recorded test points. These test points are of different locations. The test point No. 1 is around the corner, the test point No. 2 is on the straight path and the test point No. 3 is in one of the parking slots. The reference coordinates were pre-recorded by using one of the test vehicles. We then drove another vehicle to the three points five times. The localized coordinates are shown in Table 1. ∆x and ∆y indicates the difference between the test point and the reference point. ∆d is the distance between the two points, where $∆d=\sqrt{{∆x}^{2}+{∆y}^{2}}$.

The final revisiting error can be evaluated by averaging the mean errors of three test points ((0.382+0.211+0.26)/3), which is 0.284 m. This can already meet the demand of the low-speed autonomous driving in an indoor parking lot.

We also evaluated the mapping accuracy by comparing the distances between selected tag pairs in the map with the distances measured by hand using a laser measure. As shown in Table 2, five evenly distributed tag neighbor pairs, i.e., (14, 16), (4, 30), (54, 57), (19, 39) and (8, 10), are selected and their distances are measured as $d_{r}$. Based the coordinates of these tags in the map, $x_{1}$ and $y_{1}$, their distances are calucalted as $d_{c}$. ∆d is the difference between $d_{c}$ and $d_{r}$, and the mean ∆d is just 0.10 m.

The time cost of the proposed system is presented in Table 3. The total processing time for detection is around 0.2 s per frame. The optimization for the whole graph took 0.137 s at maximum since the number of vehicle node in graph grows and the processing time increases linearly.

This proposed SLAM system enables the vehicle to pass through a 3-m wide entrance and several 90-degree corners, ensuring robust localization performance inside the parking lot.

#### Comparison with Traditional VSLAM Methods

We applied the well-known ORB-SLAM2 [5] and DSO [19] methods to our parking lot both online and offline. The input of the ORB-SLAM2 system is the front-view images and the same dead-reckoning results. We employed the pre-trained vocabulary dictionary from the original ORB-SLAM2 system. Figure 1a shows examples of feature matches in ORB-SLAM2. Although the visual fiducial tags in the environment add additional textures for ORB-SLAM2, many mismatching feature pairs remain, which prohibits the initialization of the system. The original DSO fails to initialize because of the dim lighting conditions. We then turn to the stereo DSO method developed by HorizonAD (https://github.com/HorizonAD/stereo_dso). However, this method also fails in the second turn when a mild turning maneuverer was conducted, which is presented in Figure 1b. The stereo ORB-SLAM also fails in the same second turn. In addition, none of the tested methods is able to re-localize the vehicle as tested in Section 4.2.

### 4.3. How Many Visual Fiducial Tags Are Needed?

Visual fiducial tags add crucial assistance to the robust semantic SLAM system for autonomous driving. However, they may confuse human drivers and require additional work for placement. Therefore, we analysed and attempted to minimize the number of fiducial tags without affecting the mapping and localization accuracy. We extract the tag and slot cohabited area and select 36 tags for analysis. The influence of the observation frequency and distribution are discussed separately.

#### 4.3.1. Observation Frequency-Based Analysis of Tags

**Figure 14:**
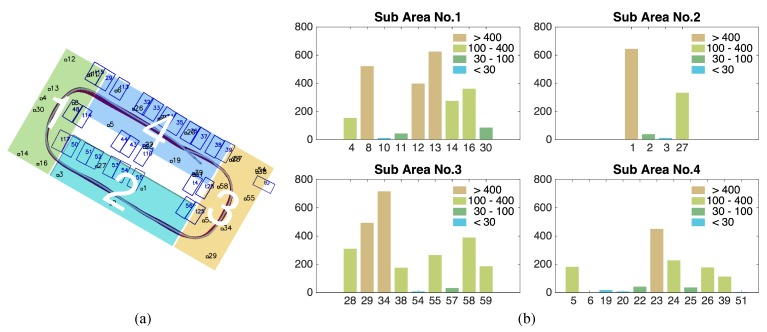
(**a**) The segmentation plan based on the vehicle trajectory and sensor’s field of view. (**b**) Observation frequency of tags in four subregions with tags of high frequency (in khaki), where the horizontal axis represents the tag IDs and the vertical axis represents the observation frequency of each tag. The frequencies can be clustered into four ranges: above 400, 100–400, 30–10 and below 30.

**Figure 15:**
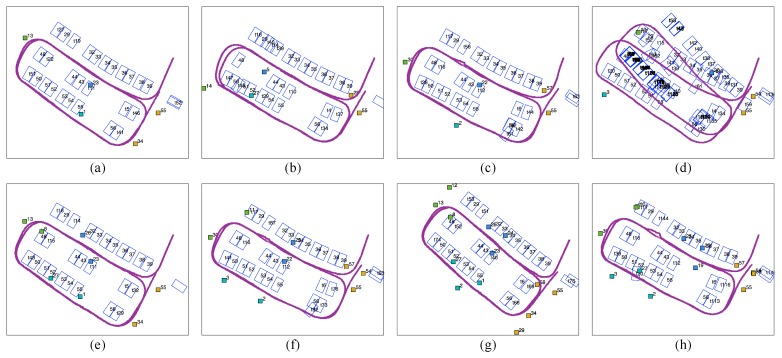
Mapping results obtained by retaining various numbers of tag with different observation frequencies. (**a**) Retaining the first four most frequently observed tags, (**b**) retaining the first four tags with high (100–400) observation frequency, (**c**) retaining the first four tags with low (30–100) observation frequency, (**d**) retaining the four least frequently observed tags, (**e**) retaining the first eight most frequently observed tags, (**f**) retaining the first eight tags with low (30–100) observation frequency, (**g**) retaining first twelve most frequently observed tags, (**h**) retaining twelve least frequently observed tags.

We divide the parking lot according to the front camera’s field of view (Figure 14a). Since the camera can only capture tags right ahead, the rectangle parking lot is divided into four sub-regions. The vehicle moves at a low, almost constant speed so that tags have an equal opportunity of being observed. The observed frequencies of all the tags are counted and clustered in Figure 14b, and each of the frequency ranges is marked in a different colour. We then activated various combinations of tags based on their observation frequency in 4 subregions and evaluated the mapping results. With a limit on the total number of tags, the numbers of tags selected from each subregion are 1, 2 and 3, and the corresponding results are shown in Figure 15.

Figure 15a–d show the results with four tags selected (one tag from each subregion). Tags with different observation frequencies (from high to low) are used in each result. In Figure 15d, where the 4 least frequently observed tags are selected, the mapping is unable to sustain global loop closure. In the mapping result for tags with low (30–100) (Figure 15c) and high (100–400) (Figure 15b) observation frequencies, some misplaced slots are present in the slot sequence, and the vehicle trajectory is bent. By contrast, the four most frequently observed tags ensure a good trajectory and slot alignment (Figure 15a). Figure 15e–h show the results with eight tags and with twelve tags selected, respectively (Restricted by the number of tags, only two groups are compared for each case). In Figure 15e,f, the eight most frequently observed tags perform well, and there are unusual and skewed parking slots and twists in vehicle trajectory when eight tags with low observation frequency are used.

In Figure 15g,h, the most frequently observed tags act as robust fiducial tags, as before. The twelve least observed tags give a better result than the four or eight least frequently observed tags. The vehicle trajectory roughly loops on the global scale although local disagreements and misplaced parking slots are observed.

As a result, in Figure 15a,e,g, where only the most frequently observed tags are used, the cycled vehicle trajectory and parking slots show the best alignment. The use of even one tag with high observation frequency in each subregion (Figure 15a) can provide reliable mapping and localization results that are comparable to the results generated with the eight or twelve tags with the highest observation frequency. Therefore, even a limited number of tags with the highest observation frequency is effective for loop closure.

To identify other factors that influence the effectiveness of the tag arrangement, the influence of the tags’ distribution is analysed based on frequently observed tags.

#### 4.3.2. Position-Based Analysis of Tags

**Figure 16:**
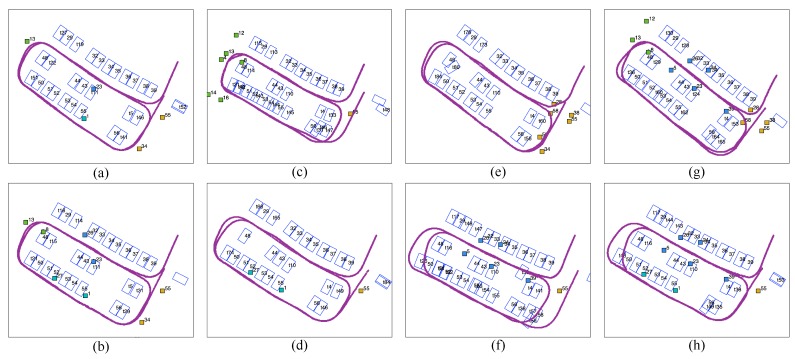
Performances of evenly distributed (**a**,**b**) and gathered (**c**–**h**) tags. All tags used here have an observation frequency greater than 100. (**a**) Retaining the most frequent observed tag from each subregion, (**b**) retaining the first two most frequently observed tags from each subregion, (**c**) retaining all six tags in subregion No. 1, (**d**) retaining all two tags in subregion No. 2, (**e**) retaining all seven tags in subregion No. 3, (**f**) retaining all five tags in subregion No. 4, (**g**) retaining twelve tags from the right-hand side of the parking lot, (**h**) retaining seven tags from subregions No. 2 and No. 4.

Since tags with high observation frequency are good fiducial tags, only frequently observed tags (with an observation frequency greater than 100) are considered in this analysis. Two groups of tag combinations are tested: tags evenly distributed in one group (Figure 16a,b), and tags located close together (Figure 16c–h). In the former group, we tested the top one or two most frequently observed tags (four or eight tags in total). In the latter group, tags are either gathered together in one of four subregions, i.e., tags from region 1, 2, 3, 4 only, or in an area of the parking lot, i.e., tags from the right-hand side of the parking lot or from a combination of regions, e.g., 2 and 4.

According to Figure 16, the mapping results with the top one or two most frequently observed tags in each subregion are the best (Figure 16a,b), where three rows of parking slots are aligned and the vehicle trajectory loops after two rounds. In the gathered group (Figure 16c–h), gathered tags are good fiducial tags for local optimization but are not reliable for the global map. In Figure 16g, the vehicle trajectory and most slot positions are optimized, although the fiducial markers are located in one lane. This is because these tags are around the global loop (where the vehicle first revisits its former trajectory), which provides robust evidence for global loop closure. However, there are small angular deviations in most slots’ directions (with one slot with extremely large angular deviation) due to the absence of local fiducial tags. Despite the effectiveness in global loop closure of the tags concentrated near the global loop, evenly distributed tags provide better fiducial markers for local slot and trajectory optimization, as well as global loop closure. Four tags, consisting of the most frequently observed tag from each subregion, are better fiducial markers than gathered tags, even when there are more tags (even twelve) in uneven situations.

#### 4.3.3. Explanation Based on Graph Configuration

**Figure 17:**
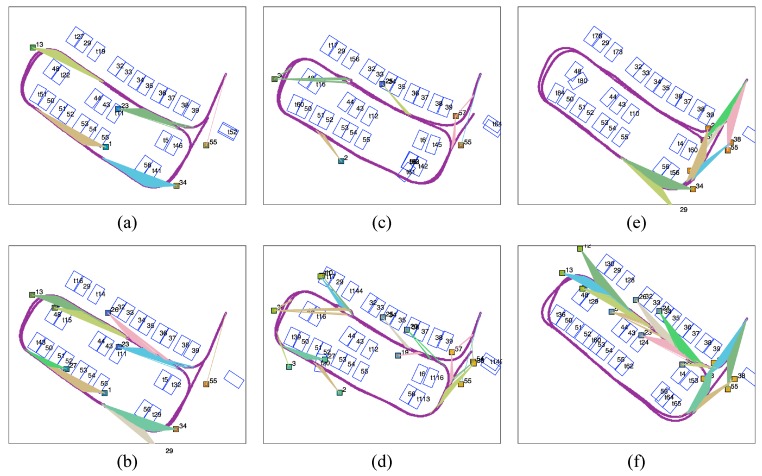
Visualization of connectivities in ’good’ maps (**a**,**b**) and ’bad’ maps (**c**–**f**). (**a**,**b**) The visualizations of Figure 15a,b, respectively. (**c**,**d**) The visualizations of Figure 15f,h, where low observation frequency leads to loop closure failure. (**e**,**f**) are the visualizations of Figure 16e,g, where the gathered tags ensure only local optimization.

To depict the underlying factors, we further investigated the topology of the graph structure built for optimization. Some of the ’good’ and ’bad’ maps in Figure 15 and Figure 16 are visualized in Figure 17.

In the upper two ’good’ maps (Figure 17a,b), which correspond to Figure 15a,b, the tag observations are evenly distributed and easily accessible, and the tags are visible for more than 50 % of the vehicle positions. By contrast, in the ’bad’ maps, tags are either too secluded to be observed (Figure 17c,d) or are serried in a specific location (Figure 17e,f). Figure 17c,d show the visualizations of the graphs for the cases of (Figure 15f,h). Although the tags are evenly distributed, the sparse tag observations do not provide sufficient constraints for loop closure. Figure 17e,f show the visualizations of the graphs for the cases of the gathered tag maps (Figure 16e,g. Dense tag observations ensure robust local loop closure near the concentrated tag region, but drift emerges gradually outside of the region because of the long-time absence of fiducial markers. These comparisons reflect the nature of the least-squares optimization for imbalanced constraints.

## 5. Conclusions

Due to the monotone texture, various illumination and dynamic conditions, a parking lot is a harsh environment for most visual SLAM methods. As a result, indoor autonomous parking is a bottleneck for autonomous driving applications. In this paper, we employed the detection of a semantic landmark, namely, the parking slot with its ID, and constructed a robust and practical semantic SLAM system for an indoor parking lot. To solve the problem of robust semantic data association, we proposed an extension of the max-mixture method by incorporating confidence level from the detection. Additionally, we attempted to determine the minimum number of visual fiducial markers to enhance the robustness and precision of the proposed system. We concluded that only the four most visible fiducial markers placed evenly in our test environment provide satisfactory mapping and localization results. This small number of markers drastically lessens the burden caused by the placement of a large number of markers and proves the practicability of the proposed SLAM methods for autonomous parking.

In future work, we aim to replace visual fiducial markers with general semantic clues, including instruction arrows or parking signs on pillars. Additionally, the influence of imbalanced constraints on graph optimization will be investigated. Finally, the joint optimization of the data associations and the poses of the vehicle and semantic landmarks will be investigated.

## Figures and Tables

**Table 1:** Online localization performance (unit: meters).

**Test Point No. 1**						**Reference**	**Mean**	**STD**
*x*	−8.404	−8.324	−8.631	−8.368	−8.172	−8.257		
*y*	7.477	7.973	7.171	7.563	7.761	7.909		
∆x	0.147	0.068	0.374	0.111	0.084		0.157	0.125
∆y	0.432	0.063	0.739	0.346	0.148		0.346	0.265
∆d	0.456	0.093	0.828	0.364	0.171		0.382	0.289
**Test Point No. 2**						**Reference**	**Mean**	**STD**
*x*	−1.947	−2.021	−2/023	−1.709	−1.695	−1.942		
*y*	9.838	9.706	9.816	9.371	9.538	9.756		
∆x	0.005	0.079	0.081	0.233	0.247		0.129	0.106
∆y	0.082	0.050	0.059	0.385	0.219		0.159	0.144
∆d	0.082	0.093	0.100	0.450	0.330		0.211	0.169
**Test Point No. 3**						**Reference**	**Mean**	**STD**
*x*	−4.896	−5.178	−5.269	−5.183	−4.924	−5.184		
*y*	5.995	6.533	5.941	6.249	6.138	6.167		
∆x	0.289	0.006	0.085	0.001	0.261		0.128	0.138
∆y	0.172	0.367	0.236	0.082	0.029		0.177	0.133
∆d	0.336	0.367	0.251	0.082	0.263		0.26	0.110

**Table 2:** Online mapping performance (unit: meters).

No.	$x_{1}$	$y_{1}$	No.	$x_{2}$	$y_{2}$	$d_{c}$	$d_{r}$	Δd
14	−48.898	7.298	16	−44.462	5.201	4.90	4.84	0.06
4	−44.169	20.138	30	−45.621	17.461	3.04	2.97	0.07
54	5.620	5.202	57	0.146	7.650	5.99	5.83	0.16
19	−14.118	6.760	39	−8.928	4.102	5.83	5.87	0.04
8	−36.820	19.263	10	−33.176	25.916	7.58	7.42	0.16
Mean ∆d		0.10		RMSE ∆d		0.05		

**Table 3:** Online SLAM time cost of each part (unit: seconds).

**Detection Time Per Frame**					
AprilTag Detection	0.055	Slot Detection	0.048	ID Detection	0.143
**Optimization Time Per Frame**					
Frame 1000	0.011	Frame 2000	0.074	Frame 3000	0.137
